# Rhinoplasty and the ‘Big Five’ Model: The Impact of Patients’ Personality Traits on Post-surgical Satisfaction Outcomes

**DOI:** 10.1007/s00266-025-05165-4

**Published:** 2025-09-08

**Authors:** Netanel Eisenbach, Matti Mizrachi, Amiel A. Dror, Majd Hajouj, Rania Faris, Ohad Ronen, Eyal Sela

**Affiliations:** 1 The Azrieli Faculty of Medicine, Bar-Ilan University, Sefad, Israel; 2 Department of Otolaryngology, Head and Neck Surgery, Galilee Medical Center, Nahariya, Israel; 3 Statistical Analysis Division, Galilee Medical Center, Nahariya, Israel

**Keywords:** Rhinoplasty, Quality of life, Personality, Big Five Model, NEO-FFI, RHINO scale

## Abstract

**Background:**

The nose plays a central role in facial aesthetics and perceptions of beauty. While personality influences the quality of life and self-perception, its effect on post-rhinoplasty satisfaction remains underexplored. This study investigates the relationship between personality traits and satisfaction with rhinoplasty outcomes.

**Methods:**

A comparative analysis was conducted between rhinoplasty patients and the general population, assessing personality traits and nasal perceptions using the Rhinoplasty Health Inventory and Nasal Outcomes (RHINO) scale pre and post-surgery, along with the NEO Five-Factor Inventory (NEO-FFI) for personality structure profiling based on the Big Five model.

**Results:**

A total of 117 rhinoplasty patients and 95 controls participated. Rhinoplasty patients, especially females, showed lower neuroticism and agreeableness but higher conscientiousness than controls. Female rhinoplasty patients had reduced negative affect and self-reproach, while males exhibited lower positive affect relative to controls. RHINO scale scores indicated post-surgery improvement in all aesthetic and functional domains. As for the relationship between personality traits and rhinoplasty satisfaction scores, significant correlations emerged between neuroticism and aesthetic satisfaction, while conscientiousness showed an inverse association with satisfaction among females.

**Conclusion:**

These findings suggest distinct personality patterns among rhinoplasty candidates compared to the general population, with specific traits linked to postoperative satisfaction. We recommend using the NEO-FFI as a preoperative screening tool to identify patients at risk of dissatisfaction and encourage further research across diverse populations to validate these findings.

**Level of Evidence III:**

This journal requires that authors assign a level of evidence to each article. For a full description of these Evidence-Based Medicine ratings, please refer to the Table of Contents or the online Instructions to Authors www.springer.com/00266.

## Introduction

Across diverse cultures and societies, the appearance of the face, especially the nose, plays a crucial component in the perception of human beauty. Studies have shown that attractive faces activate reward centers in the brain [1], and that adolescents and adults with more aesthetically pleasing appearances receive better treatment and medical care than those with less attractive appearances [2]. The question of what makes a face more attractive, and why we have specific preferences in this regard, has long preoccupied philosophers and scientists.

Personality traits can significantly impact quality of life (QOL) because they play an important role in how patients respond to different situations. This can potentially affect how patients perceive their nose structure, both before and after surgery.

The Big Five model of personality [3, 4] is one of the most widely used, validated, and well-known psychological models of personality [5]. This model assesses human personality traits across five broad domains: conscientiousness, neuroticism, extraversion, openness, and agreeableness. These traits affect how people perceive and process events, as well as how they make decisions and behave, particularly in response to significant life events.

The proposition that patients' personality traits can significantly influence their desire to undergo plastic surgery and their subsequent satisfaction with the outcomes is commonly posited.

Previous research [6–8] has investigated the personality traits of individuals seeking plastic and aesthetic surgery. However, a comprehensive comparison of the personality profiles of those seeking rhinoplasty and the general population has not yet been conducted. The existence of this research gap holds substantial importance, as it has the potential to offer valuable insights into the motivations and psychological determinants underlying the choice to undergo rhinoplasty.

To our knowledge, to date, no studies have investigated the impact of personality traits on the subjective perception of nose and face appearance, or the postoperative outcomes of plastic surgery, particularly facial plastic surgery. This study has several goals: Its primary aim is to determine whether personality structure influences post-rhinoplasty subjective aesthetic and functional satisfaction outcomes, and as a secondary goal, to delineate the personality traits of individuals seeking rhinoplasty.

## Methods

### Study Population

The study group consisted of patients aged 17 and above who underwent elective primary rhinoplasty surgery at the Galilee Medical Center between 2019 and 2022. The control sample consisted of healthy volunteers without nasal pathology, who were recruited during the same time period from the Ear, Nose, and Throat clinic of the Galilee Medical Center. These volunteers were randomly selected from outpatient clinic visitors who were not seeking treatment for nose problems, as well as hospital staff, students, and family members accompanying patients.

### Process

Two valid questionnaires were used in this study: The validated Hebrew version of the Rhinoplasty Health Inventory and Nasal Outcomes (RHINO) scale [9, 10], a short 10-item questionnaire that assesses the subjective aesthetic and functional aspects of the nose before and after surgery; and the NEO Five-Factor Inventory (NEO-FFI) [11, 12], a 60-item questionnaire that provides a comprehensive assessment of personality structure according to the Big Five model.

The RHINO scale is a validated questionnaire that assesses patient satisfaction with the functional and aesthetic outcomes of rhinoplasty [9, 10]. The scale is a 10-item questionnaire, with 5 items pertaining to aesthetic aspects (odd-numbered items) and 5 items to nasal function (even-numbered items). Each item is scored on a Likert scale from 1 to 5, with a high score indicating greater satisfaction. The 10-item scores are then added and multiplied by two to create a final score on a 100-point scale.

The NEO-FFI [3, 4, 11, 12] is based on the Big Five model, one of the most widely used and validated psychological models of personality. The NEO-FFI questionnaire consists of 60 items, with each personality trait assessed based on 12 items. The Big Five personality traits measured are neuroticism, extraversion, agreeableness, openness to experience, and conscientiousness, each of which is further divided into sub-traits. Participants responded to the questionnaire using a 4-point Likert scale, ranging from 0 (completely disagree) to 4 (completely agree).

To reduce potential bias, all patients were operated on by the same surgeon (ES). The patients completed the two questionnaires: the NEO Five-Factor Inventory (NEO-FFI) before their surgery, and the RHINO scale twice, once before their surgery and again three months after their surgery. The questionnaires were administered digitally both upon admission for surgery and three months after surgery, following a reminder sent to patients via WhatsApp.

The personality traits of the two study groups were compared, and the relationship between personality structure and improvement in the RHINO scale questionnaire following rhinoplasty surgery was examined.

### Variables

The primary dependent variable was the patient's quality of life, as measured by the RHINO scale. The independent variables were the patients' personality traits, as measured by the NEO Five-Factor Inventory (NEO-FFI); their age, sex, previous nasal surgery, and relevant health issues.

### Statistical Analysis

Data were analyzed using IBM SPSS Statistics, version 27.0. Normal distribution was assessed using the Kolmogorov–Smirnov test. For continuous variables with a normal distribution, an independent t test was used, and the Mann–Whitney U test for nonnormally distributed variables. The comparison tests were performed using a paired-sample t test and a Wilcoxon signed-ranks test. Correlation between variables was measured by Spearman's rank correlation coefficient. All tests were two-tailed unless otherwise stated. P values less than 0.05 on a two-sided test were considered statistically significant. In distributed data, values are given as mean ± standard deviation.

## Results

### Population Characteristics

A total of 117 rhinoplasty patients and 95 healthy control individuals participated in the study. The mean ± SD age of the rhinoplasty patients was 26.6 ± 8.3 (median 25) years and 33.56 ± 12.2 (median 30) for the control group, showing a minor difference between the groups, with the control group being slightly older. Sex distribution was similar between the groups, with 81.2% females in the rhinoplasty group and 76.8% in the control group (*p* = 0.386). The rate of smokers in both groups was also the same, 11.2% in the study group and 11.8% in the control group (*p* = 1.0).

### Personality Traits of the Rhinoplasty Group (Study Group)

We analyzed the rhinoplasty group's five main personality traits (neuroticism, extraversion, agreeableness, openness, and conscientiousness) compared to the control group (Table 1). We then compared the sub-traits for each trait and divided the data into males and females.

**Table 1:** NEO five-factor inventory (NEO-FFI) questionnaire scores for each personality trait of the study population

Main and Sub-traits	Study group (SG)			Control group (CG)			*P* value (SG vs CG)		
	General ( n = 117)	Females ( n = 97)	Males ( n = 20)	General ( n = 97)	Females ( n = 73)	Males ( n = 22)	General	Females	Males
Neuroticism (main)	21.65 (±9.65)	21.29(±9.87)	23.5(±8.99)	24.03(±8.70)	25.16(±8.39)	20.27(±8.82)	0.063	**0.008**	0.194
Negative affect	10.33(±4.25)	10.25(±4.26)	10.85(±4.48)	11.01(±3.81)	11.59(±3.56)	9.09(±4.07)	0.228	**0.032**	0.198
Self-reproach	11.4(±6.19)	11.15(±6.4)	12.65(±5.29)	13.02(±5.60)	13.58(±5.57)	11.18(±5.42)	**0.049**	**0.011**	0.284
Extraversion (main)	27.46 (±5.23)	28.05(±4.87)	24.9(±6.08)	27.53(±4.42)	27.32(±4.26)	28.0(±9.47)	0.924	0.306	0.095
Positive affect	8.48(±2.52)	8.81(±2.24)	7.0(±2.97)	8.88(±2.07)	8.81(±2.01)	9.14(±2.3)	0.2	0.994	**0.016**
Sociability	8.07(±2.29)	8.2(±2.26)	7.55(±2.37)	8.05(±2.19)	7.97(±2.21)	8.32(±2.17)	0.96	0.515	0.413
Activity	10.88(±2.69)	11.0(±2.71)	10.35(±2.76)	10.59(±2.45)	10.53(±2.5)	10.77(±2.33)	0.416	0.255	0.684
Openness (main)	27.28 (±5.12)	27.49(±5.38)	26.45(±6.16)	28.56(±5.07)	28.66(±5.04)	29(±5.26)	0.084	0.155	0.236
Aesthetic interests	9.28(±2.89)	9.47(±2.94)	8.45(±2.46)	9.89(±2.55)	9.93(±2.52)	9.77(±2.69)	0.108	0.29	0.096
Intellectual interests	7.89(±2.27)	7.84(±2.19)	8.25(±2.61)	7.71(±2.26)	7.64(±2.29)	7.91(±2.18)	0.558	0.569	0.592
Unconventionality	10.1(±2.88)	10.17(±2.72)	9.75(±3.68)	10.96(±2.51)	11.08(±2.43)	10.55(±2.77)	**0.024**	**0.025**	0.56
Agreeableness (main)	32.0(±5.84)	32.37(±5.63)	30.1(±6.87)	33.8(±5.82)	34.26(±5.85)	32.5(±5.57)	**0.026**	**0.035**	0.211
Non antagonistic orientation	18.34(±4.56)	18.57(±4.37)	17.15(±5.51)	20.24(±5.60)	20.62(±4.54)	19.0(±4.68)	**0.003**	**0.004**	0.241
Pro-social orientation	13.55(±2.12)	13.66(±2.23)	12.95(±2.11)	13.56(±1.81)	13.64(±1.87)	13.27(±1.58)	0.969	0.952	0.7
Conscientiousness (main)	37.19(±6.96)	37.36(±7.05)	36.25(±6.79)	33.49(±6.99)	33.37(±7.06)	33.0(±6.87)	** < 0.001**	** < 0.001**	0.236
Orderliness	14.3(±4.04)	14.39(±4.18)	13.95(±3.5)	13.01(±3.74)	12.88(±3.68)	13.45(±4.01)	**0.005**	**0.015**	0.62
Goal striving	9.57(±2.139)	9.64(±2.13)	9.15(±2.23)	8.39(±1.91)	8.37(±2.0)	8.45(±1.6)	** < 0.001**	** < 0.001**	0.3
Dependability	13.21(±2.35)	13.2(±2.39)	13.15(±2.28)	12.09(±2.61)	12.12(±2.7)	12.0(±2.37)	**0.001**	**0.007**	0.173

The bold data indicate significance (*p* < 0.05) results

The differences between the groups, indicate that rhinoplasty patients, especially females, were significantly less neurotic (*p* = 0.008), less agreeable (0.035), and more conscientious (*p* < 0.001) than the control group.

In terms of neuroticism, female rhinoplasty patients had significantly less negative affect (*p* = 0.032) and self-reproach (*p* = 0.011) than the female control group.

Male rhinoplasty patients had significantly less positive affect, sub-trait of extraversion, than the male control group (*p* = 0.016). This difference was not found among female rhinoplasty patients.

Rhinoplasty patients, especially females, were significantly less agreeable than the control group (*p* = 0.026). When divided into sub-traits, rhinoplasty patients had significantly less non-antagonistic orientation (*p* = 0.003), but there was no difference in prosocial orientation.

Finally, rhinoplasty patients, especially females, were significantly more conscientious than the control group (*p* < 0.001). This included significantly more orderliness (*p* = 0.005), goal-striving (*p* < 0.001), and dependability (*p* = 0.001).

### The Subjective Results of Rhinoplasty Surgery

A total of 117 patients who underwent rhinoplasty surgery completed the pre and postoperative RHINO scale questionnaire. The overall questionnaire score, as well as the aesthetic score (summation of 5 items of aesthetic aspects) and the functional score (summation of 5 functional items), were compared (Table 2). Scores for each item were also compared separately. All measured parameters demonstrated statistically significant improvement (*p* < 0.001) following surgery. The average improvement in all aesthetic and functional parameters after surgery was 71%. The average improvement in aesthetic parameters was 90%. Functional parameters, as measured by the questionnaire, improved by 55% after surgery.

**Table 2:** RHINO scale scores by items of the study population ( n = 117)

Item	Control group	Rhinoplasty group		Pre/post difference^+^	Rate of improvement (%)
		Pre-operative	Postoperative		
1	3.45 (±1.35)	2.01(±1.04)	4.2 (±1.12)	2.19	109
2	4.31 (±1.15)	2.5 (±1.45)	4.46 (±0.97)	1.96	78
3	3.52 (±1.31)	1.77 (±1.03)	4.22 (±1.07)	2.45	138
4	4.29 (±1.16)	2.72 (±1.45)	4.44 (±0.97)	1.72	63
5	3.73 (±1.27)	1.93 (±1.01)	3.92 (±1.24)	1.99	103
6	4.05 (±1.17)	2.51 (±1.54)	4.2 (±1.08)	1.69	67
7	3.82 (±1.05)	2.5 (±0.93)	4.31 (±0.99)	1.81	72
8	4.06 (±1.3)	2.38 (±1.56)	4.22 (±1.21)	1.84	77
9	3.89 (±1.08)	2.92 (±1.01)	4.5 (±0.89)	1.58	54
10	4.48 (±0.98)	3.99 (±1.14)	4.55 (±0.88)	0.56	14
Aesthetic score^*^ (0-50)	36.82 (±10.66)	22.26 (±6.75)	42.38 (±9.07)	20.12	90
Functional score^#^ (0-50)	42.4 (±9.74)	28.2 (±11.71)	43.74 (±8.16)	15.54	55
Total score^^^ (0-100)	79.22 (±17.04)	50.46 (±14.79)	86.12 (±14.87)	35.66	71

*Odd-numbered items represent the Rhino scale aesthetic aspects, and the aesthetic score is a summation of those items

^**#**^Even-numbered items represent the Rhino scale Functional aspects, and the functional score is a summation of those items

^+^Pre/Post difference = subtraction of the pre-rhinoplasty RHINO scale scores from the post-rhinoplasty RHINO scale scores

^**^**^ The 10-item scores are summed and multiplied by two to create a final score on a 100-point scale

We defined a satisfied rhinoplasty patient as one who gave a score of 4 (somewhat satisfied) or 5 (very satisfied) on at least 7 out of 10 items in the RHINO scale after the surgery (Table 3). This definition yielded a satisfaction rate of 76.1% (89/117) among rhinoplasty patients. The control group (healthy patients) had a satisfaction rate of 49.5% (47/95). The difference in satisfaction rates between the groups was highly significant, with a p value of less than 0.001.

**Table 3:** Satisfaction rate of the study population ( n = 117) as a calculation of the RHINO scale*

	Rhinoplasty group		Control group	
RHINO scale item	Frequency	Rate %	Frequency	Rate %
0	2	1.7	3	3.2
1	3	2.6	6	6.3
2	2	1.7	8	8.4
3	0	0	1	1.1
4	5	4.3	4	4.2
5	5	4.3	20	21.1
6	11	9.4	6	6.3
7	9	7.7	6	6.3
8	9	7.7	6	6.3
9	19	16.2	6	6.3
10	52	44.4	29	30.5
7+8+9+10	**89**	**76.1%**	**47**	**49.5%**
Total	117	100	95	100

*High satisfaction rate is define by score of 4 or 5 (meaning somewhat satisfied to very satisfied)

### Smoking and Rhinoplasty Surgery

The study group consisted of 13 smokers and 104 non-smokers. Despite the relatively small size of the smoking group, the results showed that the two groups were similar in terms of their personality traits, except for 'agreeableness'. Smokers were found to be less agreeable than non-smokers (*p* = 0.026).

We also compared the RHINO scale scores of the two groups. The results showed that smokers had lower overall scores in the pre-operative period (2-tailed *p* = 0.088, 1-tailed *p* = 0.044) and lower functional scores (2-tailed *p* = 0.051, 1-tailed *p* = 0.025). However, there was no difference in the aesthetic scores between the two groups (*p* = 0.473).

Postoperative, there was no difference in the functional (p = 0.459), aesthetic (p = 0.436), or overall scores between smokers and non-smokers.

### Relations Between Personality Traits and Subjective Improvement Following Rhinoplasty Surgery

We calculated the improvement in the RHINO scale questionnaire before and after the surgery (Table 2). Then we examined whether there is a relationship between patients' personality characteristics and the aesthetic, functional, and general improvement following the surgery.

We found a statistically significant direct relationship between the aesthetic difference and the personality trait 'neuroticism' (*r*_*S*_ = 0.185, 2-tailed *p* = 0.045, for the general population, and *r*_*S*_ = 0.199 solely for the female group, Table 4). Also, the pre-operative aesthetic perception of the neurotic patient is statistically low (*r*_*S*_ = −0.268, 2-tailed *p* = 0.003, Table 4). In other words, patients with higher levels of neuroticism tended to have lower baseline aesthetic scores, and they experienced greater improvements in the aesthetic aspect following surgery.

**Table 4:** Post-rhinoplasty satisfaction rates for each of the main personality traits, based on changes in RHINO Scale scores, pre and post procedure^#^

			Neuroticism		Extraversion		Openness		Agreeableness		Conscientiousness	
			r_S_	*P* value	r_S_	*P* value	r_S_	*P* value	r_S_	*P* value	r_S_	*P* value
General study group population ( n = 117)	Aesthetic scores	Preoperative	−0.268	**0.003**	0.028	0.763	−0.101	0.280	−0.021	0.824	0.086	0.356
		Postoperative	0.064	0.491	0.149	0.110	0.075	0.422	−0.033	0.727	−0.078	0.400
		Pre/Post difference^#^	0.185	**0.045**	0.120	0.199	0.111	0.234	−0.013	0.887	−0.112	0.230
	Functional scores	Preoperative	0.004	0.970	0.013	0.890	−0.084	0.370	0.041	0.659	−0.153	0.100
		Postoperative	−0.018	0.849	0.024	0.797	0.026	0.777	−0.065	0.485	0.020	0.833
		Pre/Post difference^#^	−0.006	0.950	0.022	0.814	0.077	0.406	−0.040	0.666	0.129	0.164
	General RHINO scale scores	Preoperative	−0.112	0.228	0.020	0.835	−0.094	0.314	0.019	0.836	−0.062	0.504
		Postoperative	0.022	0.815	0.128	0.170	0.046	0.623	−0.049	0.601	−0.013	0.887
		Pre/Post difference^#^	0.120	0.199	0.087	0.353	0.093	0.319	−0.024	0.794	0.015	0.872
Female population of the study group ( n = 97)	Aesthetic scores	Preoperative	−0.242	**0.018**	0.020	0.844	−0.079	0.447	0.023	0.829	0.154	0.137
		Postoperative	0.087	0.403	0.117	0.259	0.100	0.336	−0.099	0.342	−0.110	0.287
		Pre/Post difference^#^	0.199	0.054	0.079	0.446	0.128	0.216	−0.079	0.447	−0.190	0.065 (**1−tailed 0.033**)
	Functional scores	Preoperative	0.007	0.947	0.001	0.991	−0.115	0.267	0.036	0.729	−0.182	0.077
		Postoperative	−0.020	0.849	0.062	0.552	−0.026	0.803	−0.054	0.605	−0.003	0.977
		Pre/Post difference^#^	−0.004	0.968	0.025	0.810	0.091	0.380	0.007	0.943	0.127	0.219
	General RHINO scale scores	Preoperative	−0.097	0.348	0.017	0.873	−0.114	0.272	0.024	0.820	−0.052	0.615
		Postoperative	0.024	0.821	0.123	0.234	0.048	0.641	−0.092	0.373	−0.034	0.745
		Pre/Post difference^#^	0.124	0.230	0.066	0.528	0.127	0.221	−0.033	0.753	−0.033	0.751
Male population of the study group ( n = 20)	Aesthetic scores	Preoperative	−0.399	0.081	−0.053	0.825	−0.288	0.218	−0.120	0.614	−0.263	0.263
		Postoperative	−0.086	0.720	0.077	0.747	−0.052	0.828	0.154	0.518	0.136	0.566
		Pre/Post difference^#^	0.045	0.849	0.141	0.552	0.058	0.808	0.162	0.494	0.306	0.190
	Functional scores	Preoperative	0.013	0.957	0.071	0.767	0.084	0.726	−0.075	0.753	−0.102	0.669
		Postoperative	−0.026	0.913	−0.202	0.392	0.164	0.490	−0.239	0.310	0.129	0.587
		Pre/Post difference^#^	0.000	1.000	−0.113	0.636	−0.053	0.825	−0.181	0.444	0.220	0.351
	General RHINO scale scores	Preoperative	−0.176	0.459	0.004	0.986	0.002	0.994	−0.112	0.639	−0.182	0.441
		Postoperative	−0.021	0.929	−0.053	0.824	0.009	0.971	−0.016	0.948	0.130	0.584
		Pre/Post difference^#^	0.052	0.829	0.077	0.745	−0.045	0.850	0.003	0.991	0.321	0.168

^#^ In the RHINO scale questionnaire, odd-numbered items represent the aesthetic aspects, and the aesthetic score is a summation of those items. Even-numbered items represent the functional aspects, and the functional score is a summation of those items. Pre/Post difference = subtraction of the pre-rhinoplasty RHINO scale scores from the post-rhinoplasty RHINO scale scores

r_S_-Spearman's rank correlation coefficient

The bold data indicate significance (*p* < 0.05) results

We also found a significant inverse relationship (unilateral hypothesis) regarding conscientiousness among female patients; the more conscientious the patients are, the smaller the subjective aesthetic improvement after surgery (*r*_*S*_ = −0.190, 2-tailed *p* = 0.065, 1-tailed *p* = 0.033, Table 4).

## Discussion

Rhinoplasty ranks among the most commonly conducted plastic surgery procedures worldwide [13]. This challenging procedure aims to satisfy the patient's aesthetic and functional desires, as opposed to other non-cosmetic surgeries, which typically have the primary outcome of improving an objective medical problem. Therefore, the outcomes of this specific plastic surgery are largely subjective, depending on how the patient perceives their new nose appearance, how it meets their preoperative expectations, and how well their nose functions after the operation.

In addition to the physician's surgical skills, coordination of expectations is also important for the success of the procedure. The ability to identify unsatisfied patients preoperatively, even with good surgical outcomes, can take many years to develop [14].

The aim of our study was to investigate the subjective aesthetic and functional satisfaction rates after rhinoplasty surgery and to determine the relationship between patient personality traits and these results. We used a well-validated 60-item psychiatric questionnaire (NEO-FFI) and a 10-item combined aesthetic and functional questionnaire (RHINO scale), which were completed by both rhinoplasty patients and healthy control patients.

In this study, we found that 90% of rhinoplasty patients reported an improvement in their nose appearance, and 50% reported an improvement in their nose function. Overall, the subjective improvement was 71%. The postoperative group's RHINO scale scores were higher than or equal to the control group's scores. This suggests that the rhinoplasty surgery effectively enhanced the patients' overall quality of life.

A satisfied rhinoplasty patient was defined as one who rates 4 (somewhat satisfied) or 5 (very satisfied) on at least 7 out of 10 items on the RHINO scale after surgery. In the three months following surgery, 76.1% (89 out of 117 patients) reported satisfaction with their results. These findings suggest that rhinoplasty is a procedure that is likely to result in high levels of patient satisfaction.

Patient satisfaction outcomes, in terms of percent of satisfaction measured by Patient-Reported Outcome Measures (PROMS) rather than improvement rate measures, are not widely published in the literature. Suresh et al. [15] measured satisfaction by asking directly about functional and appearance satisfaction, reported that 82.4% of patients who underwent primary open rhinoplasty were satisfied with their results. More surgeons' experiences about their patients' postoperative satisfaction should be published to properly compare outcomes.

The psychiatric background and personality traits of aesthetic patients have been discussed in the literature for many years, including the possible effect of the patient's pre-existing psychiatric disorder on their postoperative satisfaction. It has also been suggested that the psychiatric background of aesthetic patients differs from that of the general population [16–21]. Our study supports this statement, as we found several differences in personality traits between the rhinoplasty and control groups (Table 1). Furthermore, previous studies have explored the personality traits of patients seeking different aesthetic surgeries [6, 8] or office-based aesthetic procedures [7] using the Big Five theory. These studies compared their study samples to the general population's normalized data, and not to matched controls, and identified significant specific personality traits.

Specifically, our cohort of rhinoplasty patients was found to be more conscientious, less agreeable, and less unconventional (a sub-trait of openness) than the control group. The high conscientiousness population displays a notable capacity for rational and efficient decision-making, with a high level of ambition and a dedicated pursuit of excellence. This group is notably proficient at effectively chasing their personal goals [4, 22]. Although the specific reasons for undergoing rhinoplasty were not investigated in our study, the unique characteristics of the rhinoplasty population may help to explain why they are more likely to seek plastic surgery than the general population. Considering that openness is linked to increased sensitivity to aesthetics [4], it would be reasonable to expect its higher prevalence among individuals seeking rhinoplasty; nevertheless, consistent findings indicate otherwise. Parallel to our results, Cheraghian et al. [6] and Golshani et al. 8 demonstrated comparable lower measures of the openness trait, although investigating heterogeneous aesthetic surgeries beyond just rhinoplasty patients. Interestingly, patients seeking less permanent and invasive aesthetic procedures exhibit an inverse pattern, indicating a higher openness personality trait [7]. This population also demonstrates a higher agreeable pattern, contrasting with both our findings and those of Cheraghian et al. [6]. Similar to other aesthetic population-based studies [6–8, 16–18], the study group was predominantly female (81.2%), which facilitated the statistical analysis of the female participants. In comparison with the control group, female rhinoplasty patients exhibited lower levels of neuroticism, negative affect, and self-reproach. Only male rhinoplasty patients exhibited lower levels of positive affect (a sub-trait of extraversion). Individuals with more negative affect tend to have fewer positive experiences in their lives. Additionally, people with low neuroticism may have low self-esteem [22], which may motivate them to alter their nose appearance in comparison with people with the opposite personality traits. The evidence of a higher prevalence of Body Dysmorphic Disorder (BDD) among women [20, 21] may elucidate their increased tendency toward aesthetic procedures compared to men.

The relationship between the Big Five personality traits and psychiatric disorders is a complex and evolving area of research. Some studies have found that people with low agreeableness, low conscientiousness, and low extraversion are more likely to experience mental health problems, such as depression, anxiety, personality functioning, antisocial behavior, suicidal ideation, and alcohol use [23]. For example, a study by Ruiz et al. [24] found that low agreeableness and low conscientiousness were associated with antisocial disorder and substance use disorder. Due to the difficulty in concluding from the NEO-FFI regarding psychiatric disorders diagnoses according to the Diagnostic and Statistical Manual of Mental Disorders (DSM) criteria, we encourage more research in this field.

In this study, we also investigated the association between personality traits and post-rhinoplasty satisfaction. The results showed that high satisfaction is associated with low conscientiousness (among female patients only) and high levels of neuroticism. In another report, individuals with high neuroticism tend to experience heightened levels of negative emotions and psychological discomfort, and are likely to harbor dissatisfaction with various facets of their lives [22]. Although our results are not fully consistent with the former, they can be theoretically explained by the lower baseline self-esteem associated with neurotic patients, evidenced by the lower pre-operative aesthetic scores in our sample, which may contribute to the more pronounced aesthetic improvements they experience. It is important to note that no relationship was found between satisfaction scores and ‘Openness’ main personality trait, which contains the ‘aesthetic interests’ sub-trait. Further research with larger populations may reveal a significant relationship.

Prior studies have suggested a variety of methods for preoperative screening of patients who are likely to be dissatisfied. The SAGA tool [25] offers a systemic history-taking approach that includes a list of patients' "red flags," but it does not specifically reference psychiatric disorders and leaves the decision of whether to operate on a patient up to the physician's discretion. A more recent article [26] provides a detailed assessment of the aesthetic and functional aspects of the face and nose, but it does not adequately address the mental health of the patient.

Focusing on mental parameters, several questionnaires have been suggested as pre-rhinoplasty selection screening tools. Honigman et al. [27] developed the Preoperative facial cosmetic surgery evaluation (PreFACE) questionnaire, which found that traits of depression, anxiety, low appearance evaluation, and dissatisfaction with body areas, increase the risk of dissatisfaction. However, further studies are needed to validate this questionnaire. Zojaji et al. [28] used the modified Minnesota Multiphasic Personality Inventory (MMPI) to find that obsessive and psychasthenic rhinoplasty patients were the least satisfied with the cosmetic results. However, their study did not examine the functional aspect of rhinoplasty. Babuccu et al. [16] used the full MMPI questionnaire but found no correlation to satisfaction rate. The modified MMPI, a shortened version of the MMPI, appears to be similar in terms of user-friendliness to the NEO-FFI, but further global validation is needed for the modified MMPI. It seems that there is no consensus on the best pre-rhinoplasty mental disorder screening tool, and further research is needed to develop a more objective and effective screening tool. We believe that more objective psychological measures are needed to overcome the uncertainty in patient selection. Using the NEO-FFI, we suggest that specific personality traits (conscientiousness and neuroticism) should raise additional "red flags".

This study was conducted during the COVID-19 pandemic, a time when the increased use of video conferencing and social media with self-facing cameras led to changes in facial self-image and increased demand for facial plastic surgery. In addition, there is a possible confounding effect of COVID-19 contagion on smell as an outcome of surgery, as it is well-known that COVID-19 can impair this sense, even in the long term [29]. This may explain why the improvement in smell was the lowest in the RHINO scale in our experience (14%, Table 2). Another possible explanation for the low rate of smell improvement is that the test group in this study consisted of people with primary nose problems without sinus problems, so most of them did not suffer from smell dysfunction prior to surgery.

It is well established that cigarette smoking can impair nasal breathing [30]. However, the impact of smoking on post-rhinoplasty satisfaction is a topic that has been largely neglected in the literature, and the role of smoking in satisfaction remains debatable. Elegbede et al. [31] found that smokers who underwent facial reconstruction due to trauma had lower satisfaction rates than non-smokers. In contrast, Schnabl et al. [32] reported that smoking had no impact on satisfaction among people who underwent facial reconstruction for various facial defects and tumors. In our study, we found that smokers had significantly lower baseline RHINO scale scores than non-smokers, particularly in the functional items. However, there was no difference between the two groups at the three-month follow-up. These findings suggest that smoking is not a risk factor for postoperative dissatisfaction in the short term. However, it is possible that longer follow-up or longer duration of smoking after surgery could lead to different outcomes.

Our study had several limitations. One limitation is the relatively short follow-up period. The maximum patient satisfaction is typically found 6–12 months after surgery [33]. It is possible that our study would have found different satisfaction rates if we had conducted a longer follow-up. Our three-month post-surgery routine follow-up although not entirely satisfactory, gives perspective on patients' satisfaction. Future studies can shed light with longer follow-up. Moreover, the study was carried out at one medical center following surgeries by the same surgeon (E.S.), with a relatively small number of male participants. This presents an inherent selection bias as mentioned above, given that the majority of individuals seeking this surgery at our institution are female, potentially constraining the applicability of the findings to other contexts.

Including teen and young adult patients (the youngest was 17 years old) in our cohort raises a concern of potentially influencing the satisfaction levels. Rhinoplasty is commonly performed in the late teenage years due to both functional and aesthetic concerns. Since it represents only a portion of real-world rhinoplasty candidates, the exclusion of others could limit the generalizability and clinical relevance of our findings. Existing literature on age and rhinoplasty satisfaction, as measured by the Rhinoplasty Outcomes Evaluation (ROE) questionnaire, provides mixed findings [34, 35]. Although these two studies compare patients younger and older than 30, they both include teenagers in their cohort. These conflicting results suggest that age alone may not be a definitive factor influencing postoperative satisfaction, and including younger patients allows for a more comprehensive understanding of satisfaction dynamics.

A variable that we did not include in our cohort is revision surgery. Previous surgical experiences may affect patient expectations and satisfaction. In clinical practice, rhinoplasty surgeons do encounter some revision cases, and understanding how personality traits interact with satisfaction in both primary and secondary rhinoplasty patients is crucial, but it demands a high number of cases to properly examine it statistically. More research with a relatively higher number of primary versus revision rhinoplasty candidates is suggested.

Thus, we strongly advocate for the implementation of comparable studies across diverse settings and populations. Furthermore, the available data on the personality traits of the local population is somewhat limited. While several studies have employed the Big Five model of personality to examine various influences of personality traits within the Israeli population [36–38], including gender-appropriate cohorts for our study [39], there is a lack of references regarding the indigenous personality traits of the population. To comprehensively analyze the unique personality characteristics of individuals seeking rhinoplasty, it is imperative to epidemiologically characterize the personality traits of the general population.

## Conclusions

The study demonstrates a correlation between specific personality traits and postoperative satisfaction measured by Patient-Reported Outcome Measures (PROMS). Consequently, we propose that the validated and widely recognized NEO-FFI questionnaire can potentially serve as a preoperative effective tool in identifying patients who are likely to experience post-rhinoplasty dissatisfaction.
